# A Study on Supply–Demand Satisfaction of Community-Based Senior Care Combined with the Psychological Perception of the Elderly

**DOI:** 10.3390/healthcare9060643

**Published:** 2021-05-29

**Authors:** Jiangang Shi, Wenwen Hua, Daizhong Tang, Ke Xu, Quanwei Xu

**Affiliations:** School of Economics and Management, Tongji University, Shanghai 200092, China; 84067@tongji.edu.cn (J.S.); 2030380@tongji.edu.cn (W.H.); xukeup@tongji.edu.cn (K.X.); xuquanwei613@tongji.edu.cn (Q.X.)

**Keywords:** community-based senior care, supply–demand satisfaction, SEM, psychological perception

## Abstract

Based on Maslow’s hierarchy of needs theory and customer satisfaction theory, we constructed a satisfaction model for supply–demand satisfaction for community-based senior care (SSCSC) combined with the psychological perspective of the elderly, and four dimensions of basic living needs (BLNs), living environment (LE), personal traits (PTs), and livability for the aged (LA) were selected to construct the model. The data were obtained from 296 questionnaires from seniors over 50 years old (or completed by relatives on their behalf, according to their actual situation). Twenty-two observed variables were selected for the five latent variables, and their interactions were explored using structural equation modeling. The results showed that LA was the most significant factor influencing SSCSC, and it was followed by BLNs and LE. PTs did not show a direct effect on LA, but they could have an indirect effect on SSCSC through influencing BLNs and LE. Based on the current state of community aging satisfaction, we propose to establish a community elderly care service system based on the basic needs of the elderly population, providing differentiated and refined elderly care services and improving the level of aging-friendly communities. This study provides references for the government to formulate relevant policies and other supply entities to make strategic decisions and has important implications for further enhancing community elderly services to become an important part of the social security system for the elderly.

## 1. Introduction

According to the United Nations, an aging society is defined as one in which 7% of the total population is over the age of 65. The World Population Trends Report released at the 51st session of the United Nations Commission on Population and Development, states that the global population will reach 9.8 billion in 2050. Among them, the number of people over 65 years old will exceed 1.5 billion, accounting for 16% of the total population. The increasing burden of social retirement calls for a more effective and professional senior care system [1]. Community-based senior care plays an important role in actively addressing the trend of aging. In the 1960s, the outbreak of the welfare crisis made the high welfare system unsustainable, and the theory of “aging in place” was put forward by British politicians and academics [2], and the “community-based senior care” model was created. Compared with professional institutions, communities can greatly reduce the cost of senior care. Not only can they provide comprehensive services, but they can also take advantage of the benefits of aging in place and improve the sense of belonging of the elderly while enhancing social relationships in the neighborhood [3]. The social security system introduced in the UK in 1931 provided the institutional guarantee for the implementation of community-based elderly services. In the 1960s, France began to establish and gradually improve the socialized elderly system to cover the whole country with the community-based elderly service model. Countries with a high degree of aging have adjusted their population policies and taken corresponding measures to solve this serious and realistic social problem [4]. It is expected that in 2030, China will surpass Japan in the percentage of population over 65 years of age and become the country with the highest level of an aging population in the world. 

China’s current senior care service system consists of three main models: home care, community-based care, and institutional care. Among them, the government strongly advocates community-based senior care. This model can make full use of the resources of children, senior care providers, government, and community, and enhances the ability of urban communities to serve the elderly. Each city in China has established its own unique community-based senior care model. For example, Jiangan District of Wuhan has created a network service system model, with the community residential committee as the leading agency, and three types of agencies, namely, community home-based elderly care service centers, community volunteer organizations, and community health stations, providing services for the elderly in the community. Hunan Province has created a characteristic service network, integrating medical and health care at home. How to further improve the urban senior service system and make community-based senior care an effective measure to cope with the aging crisis is an urgent issue that needs to be solved.

The supply–demand satisfaction of community-based senior care (SSCSC) in this study is an extension of the community-based senior care satisfaction on the supply and demand level, and it is an overall satisfaction judgment based on the community elderly residents’ psychological demand expectations and the current supply of elderly services in their community. The psychological perceptions, here, are divided into two parts: One is the quality of the perception of the senior care services before receiving them, i.e., the intuitive quality perception of the quantity, density, and distribution of space and related facilities. The other is the perception after receiving the services, i.e., the post-use perception of the operability and age-appropriateness of various services. For a long time, scholars in sociology, psychology, and gerontology have performed considerable research on community-based senior care satisfaction, mainly focusing on the community-living environment and locational characteristics [5], social and family support [6,7], elderly service facilities [8], personal traits [9,10], and other influencing factors to build an evaluation index system and to measure elderly satisfaction in a graded manner. In conjunction with the actual study area, factors, such as external government input [11], community environment safety [12], and community environment comfort [13], can also be taken into consideration.

In summary, community-based senior care in the United Kingdom and the United States has become a mature research area due to the mature development of community care and the deeper degree of aging. In contrast, community aging in China started late, and most of the studies focus on the present development status, and there are still few empirical studies on the satisfaction of community-based senior care in China. The only quantitative studies available have small sample sizes and are community-specific, thereby not providing valid observations of the overall study area, and the findings are not very supportive of how to improve community services. Secondly, in terms of research perspectives, existing studies are mainly limited to analyzing the role of the physical, psychological, and socioeconomic attributes of older adults, while less attention is paid to community living and the supporting environment. Therefore, on the basis of existing studies, the theoretical investigation and model analysis of elderly satisfaction need to be further improved. Third, fewer scholars have conducted empirical studies on the satisfaction of community elderly care services based on the supply and demand perspective. This study begins with the perspective of psychological cognition and analyzes the current situation of the elderly, sets foot on supply–demand satisfaction of community elderly care and possible problems, and further enriches the factor theory of community elderly care, and improves the model of accurate elderly care. It has a certain reference effect on improving existing community elderly services, which is important to better meet the elderly’s needs and make community elderly care an important part of the whole of society in building an elderly security system.

## 2. Theoretical Model and Research Hypotheses

### 2.1. Maslow’s Hierarchy of Needs

With the increase in age, the physical functions of the elderly gradually decline, the metabolism decreases, sensory deficiencies occur, intellectual capacities recede, and diseases increase, and these changes affect the psychological needs of the elderly. Psychologists categorize the psychological needs of the elderly into five aspects: survival, return, longing, symbiosis, and loneliness [14]. A study supported by the Korea Welfare Panel Study on mental health factors of single elderly living alone in different age groups (i.e., young–old, mid–old, and old–old) showed that the main factors affecting the needs of elderly people were age, subjective health, relationship with family, disposable income, house ownership, and diet [15]. Zhang and Li combined Maslow’s hierarchy of needs theory with Chinese community elderly practice and elderly related policies between 2000 and 2015 and concluded that mental needs should also be taken into consideration [16], and Lau et al., through semi-structured open interviews with elderly Japanese living alone, concluded that community elderly care should also pay attention to the community activities and recreational needs of the elderly [17].

The aging needs of the elderly, due to the physiological and psychological changes, are more complex; thus, we analyzed them with the help of Maslow’s hierarchy of needs, an important theory for studying modern behavioral science. It divides human needs into five levels: physiological, security, emotional, respect, and self-actualization [18]. At a certain stage, a person may contain multiple needs, and the needs at different levels depend on each other and overlap, determining the specific behavior of the individual [19]. This study provides a detailed explanation of aged care needs in the context of this particular group. Specifically, community-based senior care should meet the basic requirements of life care and daily care at the level of physiological needs, specifically having three meals a day with balanced nutrition, a clean and tidy living environment, etc. Especially for the empty nesters and the elderly who have difficulty taking care of themselves, the level of needs involving companionship occupies a larger proportion [20]. With aging, the decline in physical functions is a risk that the elderly need to face urgently. Also, with the change in the concept of life and quality of life requirements, the elderly have a longer life expectancy for themselves, so modern elderly people pay great attention to safety needs. Apart from safety requirements for living space, such as anti-slip, anti-noise, and accessibility, they also need to be provided with adequate medical coverage and timely medical care [21]. According to the survey, more than half of the elderly expected the community to provide them with effective regular medical checkup services, and the proportion of needs, such as rehabilitation care, health management, and emergency calls for help, was also high [22]. Emotional needs are also a noteworthy level of elderly needs in recent years. With the gradual miniaturization of family size, two-person and three-person families are becoming the main types of families. Moreover, as other family forms, such as empty-nest families, are emerging [23], the needs of emotional belonging and comfort of the elderly are gradually being paid attention. Compared with young people, older adults need to obtain a sense of organizational belonging and social identity to meet social and respect needs [24]. At the level of self-actualization needs, older people are equally eager to play with their spare time and continue to contribute to society in their later years to realize their life values [25]. It can be seen that Maslow’s needs theory has direct guiding meaning for the study of community aging, and it also provides a useful reference for the construction of a supply–demand community-based senior care satisfaction evaluation index system.

In their later life, older adults may develop different needs due to the individual factors, such as personality, physical and mental status, literacy, and economic conditions, as well as different constraints in the external environment such as family, community, and social conditions. Due to the idiosyncratic nature of elderly care services, community-based senior care appears to be unable to fully match supply and demand. In the current situation of limited resources, environmental elderly resources are obviously insufficient compared with resources for service facilities, and the supply of age-appropriate elderly services has a large deficiency [26]. Moreover, community as a platform for the effective implementation of medical and elderly care integration still needs improvement for the operability and safety of health care services [27]. Some phenomena exist, such as a lack of professional services provided by relevant personnel [28]. There is still room for improvement in the field of funding [29], cultural services [30], social participation [31], a good external supporting environment [32], and policy implementation [33] for community-based senior care [34].

### 2.2. The American Customer Satisfaction Index

The theory of customer satisfaction belongs to the field of marketing and was first introduced by Cardozo in 1965 in his article Experimental Study of Customer Input, Expectations and Satisfaction [35]. Among the existing international studies on customer satisfaction models, the American Customer Satisfaction Index (ACSI) model is the most widely used.

**Figure 1:**
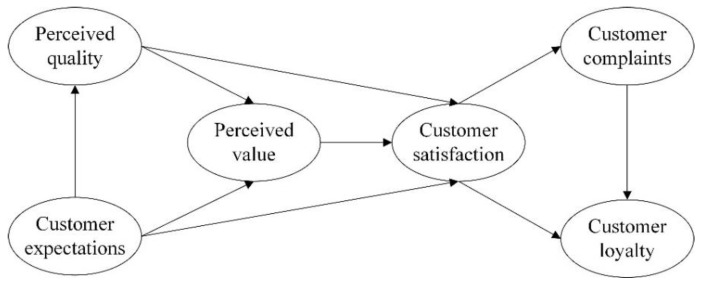
Structure of the ACSI model.

The model assumes that customer satisfaction depends on customer expectations of service quality and perceptions of quality and value, and that perceived quality, perceived value, and customer expectations are the three determinants of ACSI. After a long-term empirical study, the causal relationships among the six potential variables of the ACSI model have been confirmed as [36,37]: perceived quality, perceived value of services and products and customer satisfaction are positively correlated; customer satisfaction and customer loyalty are positively correlated; perceived quality and customer satisfaction are positively correlated; customer expectations and customer satisfaction are positively correlated; the relationship between customer expectations and perceived value is positively correlated; customer satisfaction and customer loyalty are positively correlated, and customer complaints are negatively correlated; the handling of customer complaints positively affects customer loyalty. The structure of the ACSI model is shown in Figure 1.

### 2.3. Formulation of Hypothesis

Combining the existing literature to sort out the interrelationships among variables and the understanding of the connotation of the model, we selected ACSI as the basis and adjusted the model variables according to the actual situation of community aging.

(1) Perceived quality refers to the customer’s perception of the quality of a product or service after using it, including the perception that the product meets personal needs, the perception of product reliability, and the overall perception of product quality [38]. Perceived quality plays a fundamental role in the customer satisfaction model. We investigate SSCSC based on the psychological needs of the elderly; thus, we examined perceived quality from the perspective of psychological needs. Specifically, for this study, the perceived quality is the intuitive quality perception of the number, density, distribution, and environment of the facilities related to the elderly service provider before receiving the service [39]. Combined with Maslow’s theory, human needs show a sequence from lower to higher levels, and physiological needs and safety needs are the two most basic needs of elderly people for community elderly care. Older adults have a high demand for basic care services, and the demand for medical safety increases gradually with age [40]. They need a more basic demand for the safety and accessibility of living spaces and the ability of the public environment to meet daily activities, and basic living needs (BLN) and living environment (LE) directly affect the satisfaction of older adults.

**Hypothesis** **(H1).** *BLN has a significant positive impact on SSCSC.*

**Hypothesis** **(H2).** *LE has a significant positive impact on SSCSC.*

(2) Customer expectations refer to the customer’s estimation of the quality of a product or service before purchasing and using it, where product conformity to specific needs is an important factor in determining customer expectations [40]. Customer expectations in this study refer to the heterogeneous needs of elderly people due to the fact of their age, degree of self-care, family situation, and whether they live with their spouse or children, which are related to their personal traits (PT). In fact, personal traits have a heterogeneous effect on SSCSC by influencing the needs of basic living and living environment.

**Hypothesis** **(H3).** *PT has a significant positive impact on BLN.*

**Hypothesis** **(H4).** *PT has a significant positive impact on LE.*

(3) Perceived value reflects customers’ subjective perceptions of the benefits they receive after a comprehensive assessment of the quality and price of a product or service [40]. In this study, it was defined as the perceived livability of community elderly care formed by the overall environmental conditions, facilities, location characteristics, government support, and some other compositions of the community [41]. Here, livability for the aged (LA) refers to the needs that rise to the higher level of comfort and enjoyment of life including whether the number of community elderly facilities is sufficient, whether the configuration of facilities and environment is convenient for operation, whether it meets the usage characteristics of the elderly, whether the space arrangement is reasonable, and whether the government attaches importance to the work of the elderly and so forth [42]. The demand for livability and suitability for the elderly is based on the satisfaction of basic living and housing needs and the shift of focus from “necessary” to “suitability”. It is also influenced by personal characteristics, i.e., the perception of livability is heterogeneous depending on individual circumstances [43].

**Hypothesis** **(H5).** *BLN has a significant positive effect on LA.*

**Hypothesis** **(H6).** *LE has a significant positive effect on LA.*

**Hypothesis** **(H7).** *PT has a significant positive effect on LA.*

**Hypothesis** **(H8).** *LA has a significant positive effect on SSCSC.*

**Figure 2:**
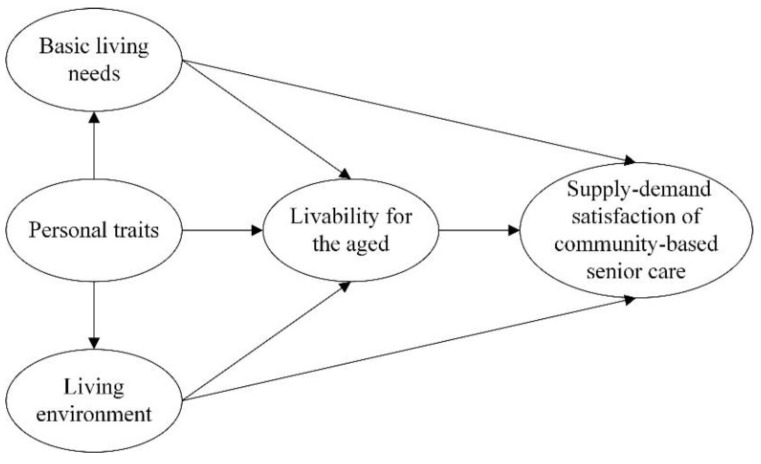
The model of the SSCSC.

Based on the selection of the model variables above, we inherited the core concept and structure of the ACSI and modified the ACSI model by combining the concept and connotation of satisfaction with the supply and demand of community aging and established the model of SSCSC by integrating the interaction among the four dimensions of BLN, LE, PT, and LA and their effects on satisfaction. Compared with the existing customer satisfaction model, we selected BLN and LE to replace the perceived quality in the original model, used PT to replace customer expectations, and chose LA to replace customer expectations. This study did not consider the customer complaints and the customer loyalty hierarchy for the time being. An SSCSC model was established is shown in Figure 2.

## 3. Research Methodology

### 3.1. Structural Equation Modeling

At present, there are various research methods on community-based senior care satisfaction, such as hierarchical analysis combined with ISM [25], multivariate analysis of variance [44], principal component analysis [45], regression analysis, and structural equation modeling (SEM), among which SEM and regression analysis are more common compared with regression analysis. SEM incorporates regression analysis, path analysis, and factor analysis, and it is a modeling tool for multivariate complex relationships.

SEM is a model for exploring, analyzing, and processing complex multivariate data based on the covariance matrix of variables, using the association between observable variables to explore the relationship among variables that are not directly observed [46]. In the social sciences, concepts, such as satisfaction [47], happiness, trust, and motivation, are not directly measurable, and other observable variables can be used as markers reflecting these unmeasurable variables to explore the relationship between unmeasurable and outcome variables.

### 3.2. Questionnaire Design

This study conducted the sample data collection required for SSCSC with the help of a questionnaire. Based on the theoretical model shown in Figure 2, 5 latent variables were considered: BLN, LE, LA, PT, and SSCSC. Combined with the previous research, 5, 4, 5, 5, and 3 observed variables were selected for these latent variables, and a model consisting of a total of 22 observed variables from the 5 latent variables was constructed for comprehensive measurement as shown in Table 1. All items measured under the latent variable were derived from previous relevant studies and were shown to be reasonable [48,49,50,51,52,53,54,55,56,57,58]. All respondents were invited to indicate their level of agreement or disagreement on a 5-point Likert scale, where 1 represents strong disagreement and 5 represents strong agreement. Before the formal distribution of the questionnaire, 3 PhD students and 2 experts in the field of management reviewed the initial questionnaire to ensure that the items in the questionnaire were set-up in a reasonable and scientific manner.

### 3.3. Data Collection and Descriptive Analysis

We designed and published a survey questionnaire based on the survey platform Questionnaire Star, using the platform’s paid sample service and inviting its registered members to fill it out. The method has been widely used in numerous previous studies [59,60]. Considering the unfamiliarity of the elderly with online questionnaires, we especially set the condition that the questionnaires could be filled out by young members of the family based on the actual situation of the elderly, i.e., the actual individual filling out the questionnaire could choose any eligible elderly person in their family as the respondent and answer the questionnaire on their behalf based on the actual situation. In the introduction to the questionnaire, we specified that the actual respondents must be over 50 years old, and the questionnaire also included the question, “Have you ever received community-based elderly care services or have you ever learned about community elderly care” in the basic information section. If the answer was, “No, I do not know”, the questionnaire was considered invalid and was not be included in the study. This was to ensure that the respondents were over 50 years old and had a certain understanding and evaluation ability concerning community elderly care. Finally, 323 questionnaires were collected, of which 296 were valid with an effective rate of 91.6%. The descriptive statistics of the respondents are shown in Table 2.

## 4. Data Analysis and Results

SEM is composed of two sets of theoretical models: the measurement model and the structural equation model. The model composed of latent and observed variables was the measurement model, which expressed the linear relationship between latent and observed variables; the model composed of exogenous and endogenous latent variables was the structural model, which expressed the linear relationship between exogenous and endogenous latent variables. The reliability and validity of the measurement model needed to be tested first in the empirical analysis to ensure the internal isomorphism of the constructed model and the degree of convergence difference among the measured variables [61]. Then, the structural model was tested for goodness-of-fit to verify the proposed hypotheses [62].

### 4.1. Measurement Model Testing

Cronbach’s internal consistency coefficient (Cronbach’s alpha) (CA) and composite reliability (CR) were used for reliability evaluation [63,64]. CA, as a widely used reliability measure, is used to assess the extent to which observed variables explain the latent variables they describe. CR is used to examine the degree of internal consistency of the corresponding items. In this paper, the values of CA and CR were calculated using SPSS 26.0. As can be seen from Table 3, the values of CA ranged from 0.605 to 0.805, which exceeds the recommended value for CA of 0.600. The minimum value of CR was 0.832, which satisfied the recommended standard of greater than 0.800 [65]. Therefore, the measurement model had good reliability and credibility.

We tested the validity by factor loadings (FLs) and average variance extracted (AVE) values of the measured variables [63]. As shown in Table 3, the AVE of the tested variables was above the minimum value of the acceptable criteria of 0.500; thus, the scale had good discriminant validity. Moreover, in Table 4, the square root of AVE for each latent variable was greater than the correlation with other latent variables, which meets the criteria of discriminant validity. According to the above analysis, the measurement model had sufficient reliability and validity.

### 4.2. Structural Model Testing

After the reliability and validity tests of the model, it was also necessary to confirm the fit of the structural model, which was processed and computed using AMOS 7.0 software. Preliminary runs revealed that the model fit did not meet the recommended criteria. Relative studies have shown that most initial models require a series of debugging to eliminate the effects of model or data errors and, thus, meet the given recommendation criteria [66]. Model modification can be accomplished by adding paths, and the path with the largest modification index (MI) (usually MI > 4 is meaningful for model modification) should be preferred for adjustment. Thus, the model was made to have a better fit by increasing the correlation of the variables under the guidance of the MI [67]. After adjustment, all the fit indices of the model met the evaluation criteria (Table 5), indicating that the empirical data had a very good fit with the assumptions of the structural equations [68,69].

**Figure 3:**
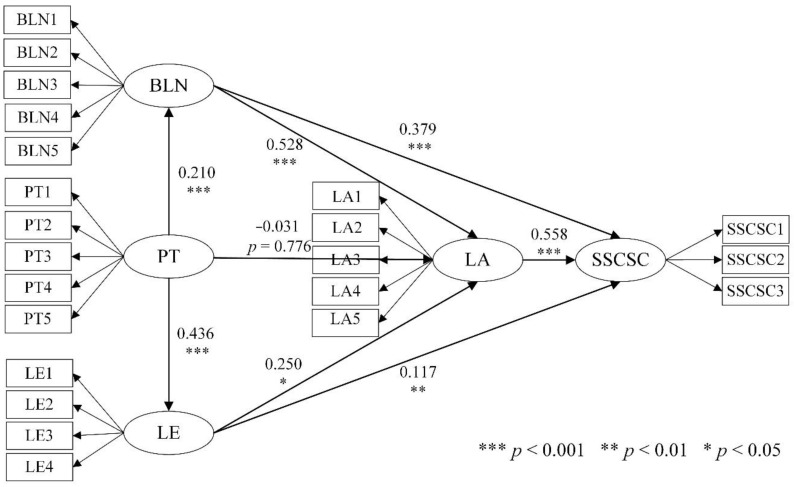
Output results of the SEM. *** Indicates significance at the 0.001 level; ** indicates significance at the 0.01 level, and * indicates significance at the 0.05 level.

The final model output is shown in Figure 3. The hypothesis validation results are shown in Table 6.

As shown in Table 6, only H7 of the original hypothesis was not supported, while the rest of the hypotheses were supported.

## 5. Discussion and Recommendations

### 5.1. Discussion of Model Results

Based on Maslow’s hierarchy of needs theory and customer satisfaction theory, we proposed a model of the SSCSC. With the basis of the elderly’s psychological perceptions and the current situation of community elderly care, an attempt was made to identify the main factors affecting the satisfaction of community senior care. By examining and analyzing the data of the 296 questionnaires collected from seniors over 50 years old or completed by relatives on behalf of seniors according to their actual situation, we obtained the following results.

(1) For BLN and LE, as the primary basic factors considered in community-based senior care, both had a more significant effect on SSCSC (BLN→SSCSC: β = 0.379, *p* < 0.001; LE→SSCSC: β = 0.117, *p* < 0.005), which supports H1 and H2.

BLN has a significant impact on SSCSC, and this result shows consistency with other related studies on the relationship between BLN and elderly care [70,71]. Lengenfelder et al. point out that with age and declining physical function, older adults have varying degrees of decline in self-care and need more care and support from outside [72]. In his study, Wang notes that older adults have a higher prevalence of chronic diseases and are more susceptible to age-related illnesses [73]. According to the Country Assessment Report on Ageing and Health in China, published by the World Health Organization in 2018, the accelerated aging of the Chinese population will lead to at least a 40% increase in the disease burden of chronic non-communicable diseases. By 2030, the number of people with one or more chronic diseases in China will more than triple. The demand for health care services among the elderly is much greater than that of the general population. Referring to the findings of Al Ketbi et al., community medical elderly services should intervene in advance to strengthen the prevention of chronic diseases in the elderly, especially those that have a greater impact on their daily lives, and to perform the health care function of community-based senior care [74]. Increasing physical activity is also an effective means of enhancing physical fitness. With the decline in the locomotor system, strengthening exercise can prevent diseases and enrich the community life of the elderly. If we categorize life and health needs as physical needs, social needs are a collection of psychological needs of the elderly. According to elderly subculture theory, community elderly care institutions are places where elderly people live together having a common activity place and similar age structure, and, subjectively, they can easily form a common area of concern, thus forming a subcultural group with a sense of identity and belonging [75]. Cultural, sports, and recreational activities are the vehicles for the formation and communication of subcultural groups that can increase a sense of belonging within members of a group, expand one’s social network, and enrich the spiritual life of the elderly. Combined with the characteristics of the psychological changes in the elderly, community care allows the elderly to live in their homes and feel the warmth of family while enjoying the space for community interaction and communication, thus increasing the satisfaction of the elderly.

Compared to BLN, the influence degree of LE on SSCSC was slightly weaker, but it was still significant. Numerous related studies have also focused on the residential needs of older adults [76,77]. Li and Li note in their study that the mental accessibility of older adults to spatial places declines with age, and a stable residential environment will help develop familiarity with the surroundings in the brain. In the context of an aging society, the planning and design of urban residential areas must take into account the physiological, psychological, and social characteristics of the elderly, and combine their behavioral trajectories and special needs for outdoor environments, etc. [78]. The community elderly environment often suffers from a lack of public space, lighting facilities, and a barrier-free design of building entrances and exits, which makes it difficult for the elderly to go out. At the same time, the intersection of pedestrian traffic and vehicular traffic, the discontinuity of sidewalks, the width, slope, road surface materials, and other problems that do not adapt to the gait characteristics of the elderly also cause difficulties for the elderly to walk [79]. Walking as a widely adopted form of activity among older adults greatly affects their community participation and, thus, satisfaction with community aging. In addition, freedom of movement is important for the physical and mental health of older adults [80]. A good community environment for leisure interaction and a positive community atmosphere are conducive to increasing older adults’ social participation and improving psychological conditions, thus enhancing satisfaction with community aging.

(2) PT had a significant effect on both BLNs and LE (PT→BLN: β = 0.210, *p* < 0.001, PT→LE: β = 0.436, *p* < 0.001), which supports H3 and H4.

Comparing the path coefficients, the effect of PTs on LE was more significant than the effect on BLNs. One possible explanation is that BLN are a more common and basic need for retirement than LE. At the level of the study where there was more differentiation in PT, the degree of the influence on LE was greater. Du et al. note that advanced aging often implies a higher risk of disability that results in a dichotomy between the increasing need for medical care among the older elderly and the increasing need for spiritual comfort among the younger elderly [81]. Lai et al. found a statistically significant positive correlation between establishing a friendly living environment and active aging through a categorical survey of 112 elderly people in Malaysia [82]. Education level, as one of the demographic variables, was also an active factor in studying the demand for elderly services. Eronen et al. investigated active aging among older adults with chronic diseases at different education levels and showed that having a certain level of health literacy was a strong predictor of active aging [83]. Literacy has different effects on the basic needs and living conditions of older adults; for example, older adults with low literacy may have a higher proportion of need for medical care, cooking, laundry, and talk-and-chat services, while older adults with high literacy have a more urgent need for health care, cultural activities, emotional confessions, and psychological counseling services. Heller and Factor’s study found that the interdependent effects of family support and intergenerational caregiving by family members can lead to better social, health, and economic well-being for older adults. Good family support includes, on the one hand, life care, but more importantly, the environment and climate of interaction in the residential setting [84]. Family support is closely related to the mental health of older adults, and older adults living alone are prone to depression, anxiety, loneliness, and other negative emotions due to the lack of support and care from their spouses in life and spirit [85]. Good family support is beneficial to reduce life stress and maintain a sense of well-being. Therefore, when studying SSCSC, we must pay attention to the influence path of PTs on BLN and LE and be concerned about the special needs of elderly people due to the fact of their age, physical quality, and illness so as to realize the refinement of elderly care services.

(3) The effect of PT on LA was not significant, which was lower than expected (PT→LA: β = −0.031, *p* > 0.05), so H7 was not supported.

Jackson and Garrett’s findings suggest that older adults’ understanding of indicators related to LA, such as accessibility to transit stops and housing conditions, was universal and consistent [86]. That is, the need for livability and aging appropriateness was universal for older adults of different ages, educational backgrounds, family support situations, and housing situations, which explains why it was less strongly associated with PT. The Report on the Development of China’s Livable Environment for the Elderly (2015), released by The National Working Commission on Aging of China, points out that, “The livable environment for the elderly is a new concept in response to the development of the aging population, and the construction of a safe, convenient and comfortable livable environment for the elderly is standard rather than high-quality mode”. The more common situation is that after retirement, the life type of the elderly gradually changes from work-oriented to leisure-oriented, with less contact with society and a narrower range of activities [87]. Older adults are more dependent on the community to sustain their health and well-being by continuing to live in the community, maintaining relationships with family and neighbors, and participating in a variety of activities in familiar places and facilities. Due to the changes in physiological and psychological status and lifestyle, the elderly need a safe and comfortable environment for aging in place, a variety of social interaction spaces in the community, and a variety of professional services in the community. The relevant government planning departments should pay attention to the general demands of the elderly to live and age well and support them, improve existing living environments, ensure the elderly truly feel that they have an environment in which to age and live well, realize the function of community livability, and make community life satisfaction more in line with actual demand.

(4) Both BLN and LE had a significant effect on LA (BLN→LA: β = 0.528, *p* < 0.001, LE→LA: β = 0.250, *p* < 0.001), which supports H5 and H6.

This view is reinforced by the study by Chiu et al. who suggest that for the elderly, food, clothing, shelter, basic medical care, and a safe and convenient living environment are the basis for quality elderly care [88]. In other words, BLN and LE were related to the basic needs of senior living, while LA emphasized the quality of senior living with peace and happiness, including the beauty of the senior living environment and the completeness of the number of related facilities. This emphasizes the improvement in the service facility system and the diversification and humanization of the content under the basic framework of health care, culture and entertainment, daily care, and senior living in the community. Gong et al. propose to improve the professionalism of elderly care services through “medical and nursing integration” so that medical and health resources and elderly care resources can complement each other to provide elderly people with adequate supply, reasonable gradients, and various forms of elderly health care services in the process of aging; thus, improving the overall capacity and level of elderly care services [89]. By exploring the utilization pattern of community elderly facilities, Chang et al. suggest that the functional configuration of elderly facilities should be refined from the perspective of demand to improve the utilization rate of facilities, enhance the safety and comfort of facilities, and improve the efficiency of facility services [90]. From the abovementioned studies, we can see that a “people-oriented” view to improve the quality and effectiveness of senior care services was fully recognized. The construction and improvement of high-satisfaction community senior care services need to start from the basic needs of senior care and consider the quantity to meet needs but also to provide high-quality community senior care services that are suitable for the elderly.

(5) LA had a significant effect on SSCSC (LA→SSCSC: β = 0.558, *p* < 0.001), which supports H8.

Currently, there are structural changes in the elderly population, and the number of people who are willing and able to lead a high quality of life in old age is gradually increasing, and the conditions, perceptions, and lifestyles of the elderly are quietly changing. The path coefficient also shows that LA was the most critical variable affecting SSCSC. In reality, the demands of the elderly for senior living was mainly reflected in two ways: one was to meet the physiological needs, mainly referring to meeting the characteristics of the elderly activities and behavior, such as adequate facilities which are easy to operate and reasonable space layout; the second is to meet the psychological needs of the elderly, which make they feel a sense of security, comfort as well as respect and care in the space. This is supported by a study by Carmody et al., who suggest that community care should further promote the level of geriatric care practice by improving the level of care and enhancing aging appropriateness [91]. Integrating research in landscape, environmental behavior, psychology, and medicine, Rajagopalan et al. suggest that natural landscapes have the effect of reducing stress, relieving emotions, preventing hidden illnesses, and restoring health [92]. Related studies have also shown a positive correlation between the amount of green space in the living environment and health [93]. In other words, the beauty of community environment affects both the physical health and psychological health of the elderly thus affecting satisfaction. At the same time, community aging cannot be achieved without the support of the government and the community. The development of a complete social security system, the provision of pension and health insurance policies as well as the specification requirements for community aging, etc., these aspects of support determine, to some extent, the quality of institutions, thus directly or indirectly affecting the perceptions of elderly people towards community aging [94]. With sufficient government support, a sound social security system, and reasonable pension levels, the elderly have enough ability to cope with illness and pay for their old-age expenses. In the face of standardized and professional community elderly care services, the elderly can enjoy more comprehensive and complete care, so the willingness and satisfaction of community elderly care increases.

### 5.2. Suggestions

Based on the above findings, this study makes the following recommendations to improve the supply–demand satisfaction of community-based senior care.

(1) Based on the basic living needs of the elderly, establish a community elderly service system that meets needs.

How to accurately grasp the needs of the elderly is an important basis for improving community elderly care services. Focused on the basic needs of the elderly for daily care and health, community elderly care service personnel should provide “warm” services with “love, patience and care” according to the special characteristics of the service recipients while improving the professionalism of nursing care, making seniors feel at home in community care. Actively promoting the development of medical and elderly care integration and exploring new models of cooperation between medical institutions and senior care institutions is an important issue in the construction of the current senior care service system. Communities can establish partnerships with professional medical institutions to build a health care service structure consisting of multidisciplinary teams. Adhering to a people-oriented ideology, medical services should be provided closely around the needs of the elderly, and appropriate health care plans should also be developed for them through daily diagnosis and comprehensive assessments. The beneficial exploration in the field of health care integration helps to further improve the satisfaction of community elderly care. The community can also improve the interpersonal relationships of the elderly, actively eliminate possible negative emotions in aging, improve the mental health of the elderly, and create a harmonious, mutually supportive and positive atmosphere by organizing highly participatory and interactive senior activities and cultural and recreational programs. In addition, the spiritual life of the elderly can be enriched by inviting volunteer services into the community and other forms to actively intervene and reduce their loneliness and improve their satisfaction with community aging. The government and the community should establish a senior care service system that meets the needs of the elderly based on the actual community senior care, with the supply side as the guide. Consider the quantitative allocation of senior care services, especially the supply of health care services, while focusing on the safety needs of the elderly, to improve the allocation of senior care resources and avoid the phenomenon of mismatch between supply and demand.

(2) Pay attention to the heterogeneous needs of individuals and provide differentiated and refined elderly care services.

Community elderly care should closely follow the actual situation, widely understand and further filter and screen needs, and summarize the general rules of different types of elderly people. Most of the needs of the elderly at the same age are homogeneous, which is consistent with the threat of physical decline and health risks. However, elderly people have different heterogeneous needs due to the fact of their different family support, education levels, and personality preferences. Therefore, for general needs, it is necessary to provide a wide range of services based on the improvement of service suitability and refinement. At the same time, we need to take into account the heterogeneous needs of the elderly and provide segmented services. For example, for empty nesters and elderly people with insufficient family support, more attention should be paid to the guarantee of life care and spiritual comfort services. For younger elderly people, the community should provide abundant recreational activities as much as possible, while for the older elderly people, the community should increase the number of care services provided and improve the professionalism of care. The government can commission relevant third parties to conduct comprehensive research on the needs of the elderly and grasp the current situation related to the different types of elderly people. In addition, interviews or questionnaires can be conducted with the elderly in the community to obtain the actual needs of the elderly in the role of service providers. At the same time, information technology can be used to establish an information database for the elderly and create an effective file to record specific information about senior care services. Communities can conduct data analyses on these needs through the database and dynamically track senior satisfaction to assess service quality. Through the stages of pre-service research, in-service inquiry and post-service feedback, the community can dynamically manage the service quality of elderly care, pay attention to the effective subjective perceptions of the elderly in the process of using the service, establish an information feedback mechanism and service problem solutions, and improve the sustainability of the service operation.

(3) Integrate the layout of community public space and improve the community elderly care environment.

The unevenness of the current external living space of the environment of aging communities limits the diversified needs of the elderly, and the previous study found that there were generally problems such as small-scale public spaces, poor openness, and incomplete facilities with a lack of barrier-free environments in current community elderly service spaces. The construction of community public space should be re-integrated and laid out to enhance community livability and the level of the age-friendly living environment. Since elderly people have limited acting ability, a small activity range, and fixed activity location, multi-level public spaces should be planned according to the activity circle of elderly people to facilitate different outdoor activities such as walking, resting, chatting, and playing chess. For different types of aging communities, the community environment should also be differently configured and improved. For example, for aging communities with dense buildings and severe aging facilities, small public green areas can be built using open spaces between houses to improve the greening level of the community. Public passages and public spaces should be renovated for barrier-free facilities. For aging communities with a good community environment and public green space, on the one hand, the greening configuration and maintenance level of public green space can be improved, on the other hand, the openness of public green space can be improved by supporting the construction of leisure and fitness facilities and increasing outdoor activity space. For new elderly residential areas, they should not only ensure the land space of the community public green space, but also support the community square, small garden, and other public space. At the same time, the design of walking paths, squares, public green areas and public facilities should be fully implemented in the design of a barrier-free walking environment. All parts of the community elderly environment facilities should be interrelated and interact with each other to form an organic whole, to give full play to its role as the basic unit of elderly services and improve the overall level of satisfaction of the community elderly.

(4) Realize substantial community livability and aging-friendly transformation.

At present, most elderly people still live in old communities, and research shows that the lack of “quantity” and “quality” of community public facilities and public spaces, such as elderly care, education, and culture, restrict the development of community elderly care services. Therefore, the transformation of this old space is an important measure to complete the improvement in community elderly care patterns. As China has paid more attention to the stock planning in recent years, more renovations have been made to the existing communities in urban areas, but more focus is paid on the energy-saving renovations and pipeline improvements in old neighborhoods, lacking systematic sorting and in-depth research on the physiological and psychological needs of the elderly, which cannot create a spatial environment truly suitable for the elderly to live and work. Therefore, we should pay attention to the quality of the current community elderly service facilities and the creation of the community environment for the elderly. For example, with the help of “Internet Plus”, a new community management model can be established to create a wise, safe, and efficient intelligent community for the elderly. It provides health management, remote care, catering reservation, online elderly assistance, and emergency assistance services for the elderly. It improves management efficiency, achieves remote monitoring and real-time responses, keeps track of the physical and mental health of the elderly, learns about sudden diseases and takes preventive measures as early as possible. Through wireless network and cloud monitoring, the community can monitor the elderly’s outdoor activities and provide timely assistance in case of sudden falls and other conditions. The elderly can also enjoy medical and shopping services without having to leave home, which is convenient and fast.. It is important to emphasize that age-friendly renovation should start from meeting the needs of the elderly, paying special attention to the psychological needs, and be realized through universal design principles.. This will make community-based care an important part of pension system reform as a complementary pension structure system.

## 6. Conclusions and Limitations of the Study

Based on Maslow’s hierarchy of needs theory and customer satisfaction theory, this study combined with the psychological perception of the elderly, established a model, and analyzed the relevant influencing factors, finally obtaining the following meaningful findings. First, the research results reflect the applicability of the constructed theoretical model. The model was based on the four dimensions of BLNs, LE, PTs, and LA to study their influence on each other and on the measured satisfaction. Seven out of 8 hypotheses were verified to be valid, the influence paths existed significantly, and the results had strong predictions and explanations. Secondly, considering the path coefficients, the results showed that the degree of LA was the most important factor, and, in addition, we should return to the bottom needs and focus on the senior care needs for basic life care and living environment. As the basis of Maslow’s hierarchy of needs, special attention should be paid to the needs of the elderly, due to the fact of physiological and psychological changes, and then improve the community care satisfaction. PTs did not show a direct effect on LA, but they could have an indirect effect on satisfaction by influencing the BLNs and LE, and the effect of PTs on LE was more significant than the other. The BLNs significantly influenced the need of LA compared to the need of LE. Therefore, while paying attention to the “quantity” of community elderly service space, we should also pay attention to the “quality” of improvement and the livability and suitability of community elderly care. In addition to the common basic needs, we should also notice individual needs, including psychological and family atmosphere factors, so as to further improve the quality of community elderly care. Based on the influence, shortcomings, and possible improvement directions of the four dimensions, the SSCSC will be improved in a targeted manner. This study has initially grasped the current development status and shortcomings of the community elderly service system through the research, which can provide a reference for the improvement and further promotion of the community elderly service, provide ideas and directions for transformation, and offer a guide for the government to formulate active and effective policies and other supply entities to make strategic decisions. It is of great significance to promote community elderly care as an important part of the old-age security system in the context of aging.

This study discusses the factors influencing the satisfaction of community aging and draws some informative conclusions, but there are also the following research limitations. First, this study used a paid questionnaire to examine SSCSC, and the data used were not obtained through community participatory research visits; instead, an online questionnaire was used and the questionnaires were collected allowing family members to fill it out on behalf of the elderly, according to their actual situation. Thus, the results of the questionnaire reflect the collective situation; thus, there may be some deviations from actual behavior [95]. Future research can consider providing more supplementary and supporting data for the study based on field research through community interviews and other forms. Secondly, this study was based on relevant theoretical modeling and examined interactions and the relationship of their effects on satisfaction from four dimensions. However, due to the factors, such as insufficient understanding of the theoretical connotation or bias in the selected observed variables, there was the situation that the explanatory power of the sample of the hypothesis construct was not strong enough, and the original hypothesis was not fully supported. Therefore, future research can further consider the study of such factors and configurations to explore the supply and demand characteristics of community elderly service spaces. Finally, this study was conducted in China in April 2021, and the results of the research data were obtained within China. Due to the factors, such as cultural background, environment, and social development stage, there may be differences between the experimental results and the actual situation in other countries or regions. For future studies, further expansion of the sample size and the regional scope of the study can be considered to obtain more generally applicable conclusions. The effect of regional differences on retirement satisfaction is also a meaningful topic to study.

## Figures and Tables

**Table 1:** Survey items related to the SSCSC.

Latent Variable	Observed Variable	Item	Related Documents
BLN	Daily Living	I think the daily living facility system (restrooms, bathrooms, dining rooms, etc.) affects the supply–demand satisfaction of community-based senior care.	[48]
	Health Care	I think the community health care facility system (health care room, rehabilitation training room, etc.) affects the supply–demand satisfaction of community-based senior care.	[49]
	Health and Fitness	I think the health and fitness facilities system (ping pong room, gym, etc.) affects the supply–demand satisfaction of community-based senior care.	[50]
	Recreation	I think the community recreational facilities system (chess room, multi-purpose room, audio–visual room, etc.) affects the supply–demand satisfaction of community-based senior care.	[51]
	Culture and Education	I think the cultural and educational facilities system (reading room, painting and calligraphy room, classroom, etc.) affects the supply–demand satisfaction of community-based senior care.	
LE	Public Activity Space	I think the overall layout of the public activity space (size, density, accessibility, etc.) affects the supply–demand satisfaction of community-based senior care.	[52]
	Community Road	I think the community road conditions (path quality, directional guidance, etc.) and resting facilities (shade from the sun and rain, etc.) affect the supply–demand satisfaction of community-based senior care.	[53]
	Community Environmental Safety	I think the community security and safety of the night environment affect the supply–demand satisfaction of community-based senior care.	
	Community Engagement Environment	I think the atmosphere of gathering activities, mutual help and harmony among community residents affect the supply–demand satisfaction of community-based senior care.	[54]
LA	Number and Configuration of Facilities	I think the distribution of the number and density of elderly service facilities in the community affect the supply–demand satisfaction of community-based senior care.	[55]
	Aging-Friendly Facilities	I think that the ease and safety of operation for the elderly service facilities in the community affect the supply–demand satisfaction of community-based senior care.	
	Environmental Beauty	I think the public activity space layout, the scenery along the road, and the environment of internal facilities in the community affect the supply–demand satisfaction of community-based senior care.	[56]
	Aging-Friendly Environment	I think the detailing of resting platforms, positioning instructions, and emergency call buttons in community activities affect the supply–demand satisfaction of community-based senior care.	
	Government Input	I think the government’s financial investment and level of attention in community care affect the supply–demand satisfaction of community-based senior care.	[57]
PT	Age	I think the age of the respondent affects the supply–demand satisfaction of community-based senior care.	[58]
	Level of Care	I think the respondents’ ability to take care of themselves and the degree to which they need to be cared for affect the supply–demand satisfaction of community-based senior care.	
	Family Support	I think the financial and emotional support of the family affect the supply–demand satisfaction of community-based senior care.	
	Education	I think the education level of the respondents affects the supply–demand satisfaction of community-based senior care.	
	Companionship	I think the respondents’ living style (living alone, living with spouse, living with adult children, etc.) affects the supply–demand satisfaction of community-based senior care.	
SSCSC	My overall satisfaction with current community-based senior care services is high.		
	Compared to what I expected, my overall satisfaction with current community-based senior care services is high.		
	Compared to my ideal level, my overall satisfaction with current community-based senior care services is high.		

**Table 2:** Basic information of the survey respondents.

Characteristics	Demographic Variable	Size	%
Gender	Male	162	54.73
	Female	134	45.27
Age	50–59 years old	123	41.55
	60–69 years old	69	23.31
	70–79 years old	82	27.70
	Over 80 years old	22	7.43
Education	Primary School and Below	41	13.85
	Junior High School	55	18.58
	High School	80	27.03
	Specialized Degree	55	18.58
	Bachelor’s degree or above	65	21.96
Income (RMB)	<3000	72	24.32
	3000–3999	51	17.23
	4000–4999	45	15.20
	5000–5999	62	20.95
	≥6000	66	22.30
Living Situation	Living alone	31	10.47
	Living with spouse	161	54.39
	Living with their children	103	34.80
	Others	1	3.38

**Table 3:** Reliability and validity analysis index values.

Latent Variable	Observed Variable	Mean	SD	FL	CA	CR	AVE
BLNs	BLN1	3.42	0.867	0.698	0.762	0.841	0.515
	BLN2	3.29	1.114	0.69			
	BLN3	3.35	1.030	0.736			
	BLN4	3.63	0.966	0.709			
	BLN5	3.41	1.019	0.753			
LE	LE1	3.34	0.951	0.724	0.605	0.832	0.553
	LE2	3.43	1.079	0.754			
	LE3	3.87	0.974	0.754			
	LE4	3.58	0.905	0.741			
LA	LA1	3.20	0.932	0.793	0.801	0.864	0.560
	LA2	3.56	1.035	0.721			
	LA3	3.70	0.898	0.719			
	LA4	3.42	1.066	0.740			
	LA5	3.39	1.011	0.765			
PTs	PT1	3.53	0.998	0.707	0.681	0.833	0.501
	PT2	3.93	1.076	0.761			
	PT3	3.84	0.960	0.694			
	PT4	3.42	1.088	0.604			
	PT5	3.92	0.935	0.761			
SSCSC	SSCSC1	3.48	0.848	0.804	0.805	0.885	0.720
	SSCSC2	3.32	1.035	0.854			
	SSCSC3	3.17	1.087	0.886			

**Table 4:** Results of the discriminant validity analysis.

Variables	BLN	LE	LA	PT	SSCSC
BLN	0.818				
LE	0.806	0.744			
LA	0.603	0.677	0.748		
PT	0.192	0.267	0.183	0.501	
SSCSC	0.661	0.657	0.699	0.110	0.849

**Table 5:** Structural equation model goodness-of-fit tests.

Fitting Index	CMIN/DF	CFI	RMSEA	GFI	AGFI	SRMR	NNFI	AIC
Results	2.173	0.860	0.075	0.858	0.817	0.107	0.832	506.000
Criteria	<3	>0.8	≤0.08	>0.8	>0.8	<0.05	>0.8	/

**Table 6:** Standard regression path coefficients of the SEM and hypothesis validation results.

Hypotheses	Path	Path Coefficients	T-Value	Significance	Hypothesis Supported
H1	BLN→SSCSC	0.379	4.512	***	Yes
H2	LE→SSCSC	0.117	2.985	**	Yes
H3	PT→BLN	0.210	3.709	***	Yes
H4	PT→LE	0.436	3.960	***	Yes
H5	BLN→LA	0.528	5.643	***	Yes
H6	LE→LA	0.250	2.412	*	Yes
H7	PT→LA	−0.031	−0.284	Insignificant	No
H8	LA→SSCSC	0.558	9.937	***	Yes

*** Indicates significance at the 0.001 level; ** indicates significance at the 0.01 level, and * indicates significance at the 0.05 level.

## Data Availability

Not applicable.
